# Effects of Moxifloxacin on Human Neutrophil and T-Lymphocyte Functions *in Vitro*

**DOI:** 10.3390/ph3123570

**Published:** 2010-12-13

**Authors:** Moliehi Potjo, Riana Cockeran, Annette J Theron, Charles Feldman, Ronald Anderson

**Affiliations:** 1 Medical Research Council Unit for Inflammation and Immunity, Faculty of Health Sciences, University of Pretoria and Tshwane Academic Division of the National Health Laboratory Service, Pretoria, South Africa; E-Mails: riana.cockeran@up.ac.za (R.C); atheron@up.ac.za (A.J.T); ronald.anderson@up.ac.za (R.A); 2 Division of Pulmonology, Department of Internal Medicine, Charlotte Maxeke Johannesburg Academic Hospital and Faculty of Health Sciences, University of the Witwatersrand, Johannesburg, South Africa; E-Mail: feldmanc@medicine.wits.ac.za (C.F.)

**Keywords:** calcium, CD25, elastase, *N*-formyl-L-methionyl-L-leucyl-L-phenylalanine, interleukin-2, phorbol myristate acetate, mitogen-activated proliferation, reactive oxygen species

## Abstract

Moxifloxacin is useful in the treatment of respiratory infections, including community-acquired pneumonia, and also shows promise in the treatment of tuberculosis, a clinical setting which necessitates extended administration of this agent. Relatively little is known, however, about the effects of this agent on the antimicrobial and proliferative activities of human neutrophils and T-lymphocytes, respectively. In the current study, we have investigated the effects of moxifloxacin at therapeutic concentrations and greater (1–20 µg/mL) on cytosolic Ca^2+^ fluxes, generation of antimicrobial reactive oxygen species (ROS), and release of the primary granule protease, elastase, following activation of the cells with the chemoattractant, fMLP (1 µM), or the phorbol ester, PMA (25 ng/mL), using radiometric, chemiluminescence, and colourimetric procedures, respectively. The effects of moxifloxacin on mitogen-activated proliferation of T cells and expression of the interleukin-2 (IL-2) receptor (CD25) were measured using radiometric and flow cytometric procedures respectively. With the exception of elastase release, which was significantly increased (P < 0.05) by treatment of the cells with moxifloxacin at 10 and 20 µg/mL, none of the other neutrophil or lymphocyte functions was affected by moxifloxacin. These observations suggest that extended use of this agent is unlikely to compromise the protective functions of neutrophils and T-lymphocytes and may even potentiate neutrophil-mediated antimicrobial activity by increasing the release of elastase.

## 1. Introduction

Most antibiotics interact cooperatively with the innate and adaptive immune defences of the infected host by weakening microbial pathogens, thus rendering them vulnerable to immune-mediated elimination [1]. In some settings, however, especially in the case of bactericidal antimicrobial agents, antibiotic-mediated disintegration of bacteria results in the release of pro-inflammatory cell wall components and intracellular toxins, posing the risk of inflammation-mediated damage to host tissues [2]. Notwithstanding, these direct interactions between antibiotics and bacteria, some classes of antibiotic, albeit relatively few, possess anti-inflammatory properties, mainly beneficial, which are achieved via their direct interactions with cells of the host immune system. Included in this group are macrolides, imidazole anti-mycotics, tetracyclines, and possibly fluoroquinolones [2]. These agents have been reported to target the production of pro-inflammatory cytokines/chemokines, matrix metallo-proteinases, leukotrienes, reactive oxygen species, expression of adhesion molecules, and to induce apoptosis [2].

Fluoroquinolone antibiotics are frequently used in the management of respiratory tract infections. In the case of community-acquired pneumonia many guidelines, such as those of the Infectious Diseases Society of America/American Thoracic Society (IDSA/ATS), consider fluoroquinolone monotherapy to be a suitable alternative to beta-lactam/macrolide combination therapy in the treatment of the sicker, hospitalized cases [3]. Furthermore, in tuberculosis (TB) including cases of drug resistant infection, fluoroquinolone antibiotics especially moxifloxacin play a major role in therapy and may be administered over a prolonged period of time [4,5,6]. However, relatively little is known about the effects of moxifloxacin on host defences, particularly innate protective mechanisms involving neutrophils, as well as the proliferative responses of T-lymphocytes.

In the current study, we have investigated the *in vitro* effects of moxifloxacin on the antimicrobial and proliferative activities of isolated human blood neutrophils and T-lymphocytes, respectively.

## 2. Materials and Methods

### 2.1. Chemicals and Reagents

Moxifloxacin was kindly supplied by Bayer Healthcare AG (Leverkusen, Germany). Moxifloxacin was dissolved in sterile distilled water and used in the assays described below at final concentrations of 1–20 µg/mL. Unless otherwise indicated, all other chemicals and reagents were purchased from the Sigma Chemical Co. (St Louis, MO, USA). This concentration range was based on previous pharmacokinetic studies which documented peak serum concentrations of 6.6–10 µg/mL, following ingestion of a single 400 mg tablet of moxifloxacin by adult humans [7,8,9].

### 2.2. Neutrophils

Permission to draw blood from healthy, adult human volunteers was granted by the Faculty of Health Sciences Research Ethics Committee of the University of Pretoria and informed consent was obtained from all participants. Purified human neutrophils were prepared from heparinised blood (5 units of preservative-free heparin/mL of blood). Neutrophils were separated from mononuclear leukocytes (MNL) by centrifugation on Histopaque^®^-1077 cushions at 400 g for 25 min at room temperature. The resultant erythrocyte/neutrophil layer was sedimented with 3% gelatin for 15 min at 37 °C to remove most of the erythrocytes. Following centrifugation (280 g at 10 °C for 10 min), residual erythrocytes were removed by selective lysis with 0.84% ammonium chloride at 4 °C for 10 min. The neutrophils, which were routinely of high purity (>90%) and viability (>95%), were resuspended to 1 × 10^7^ cells/mL in phosphate- buffered saline (PBS, 0,15 M, pH 7.4) and held on ice until use.

### 2.3. Oxidant Generation

The generation of superoxide and oxidants derived from the MPO/H_2_O_2_/halide system, were measured using lucigenin (bis-*N*-methylacridinium nitrate) and luminol (5-amino-2,5-dihydro-1,4-phthalazinedione)-enhanced chemiluminescence (LECL) methods, respectively [10]. Neutrophils (1 × 10^6^ final) were preincubated for 15 min in 900 µL indicator-free Hanks balanced salt solution (HBSS, pH 7.4, Highveld Biological, Johannesburg, South Africa) in combination with 0.2 mM lucigenin or 0.1 mM luminol in the presence and absence of moxifloxacin (1–20 µg/mL, final), prior to activation with phorbol12-myristate 13-acetate (PMA, 25 ng/mL final). Spontaneous and PMA-activated LECL responses were recorded using an LKB Wallac 1251 chemiluminometer (Turku, Finland) and the readings were recorded as mV/sec. Additional experiments were performed, in the same manner, to investigate the effect of moxifloxacin on the LECL responses of neutrophils activated with the synthetic chemotactic tripeptide *N*-formyl-L-methionyl-L-leucyl-L-phenylalanine (fMLP, 1 µM, final) using a Lumac Biocounter^®^ M2010 (Lumac Systems, Schaumberg, The Netherlands). LECL readings were integrated for 10-sec intervals and recorded as relative light units (r.l.u). The automated LKB system was unsuitable for use with fMLP-activated cells because of the rapidity of the peak response (within 30–60 seconds).

### 2.4. Elastase Release

Neutrophil degranulation was measured according to the extent of release of the primary granule-derived protease, elastase. Neutrophils (1 × 10^6^ cells/mL final) suspended in HBSS in the presence and absence of moxifloxacin (1–20 µg/mL) was incubated for 10 min at 37 °C. The stimulant fMLP (1 µM final) in combination with cytochalasin B (CB, 1 µM) was then added to the cells, which were incubated for 15 min at 37 °C, after which the tubes were transferred to an ice bath, followed by centrifugation at 250 g for 10 min to pellet the cells. The neutrophil-free supernatants were assayed for elastase activity using a micro-modification of a standard spectrophotometric procedure [11]. Briefly, 125 µL of supernatant fluid was added to 125 µL of the elastase substrate *N*-succinyl-L-alanyl-L-alanyl-L-alanine-*p*-nitroanilide, 3 mM in 0.05 M Tris-HCl (pH 8.0). Elastase activity was monitored at the wavelength of 405 nm using a Power Wave_X_ microplate spectrophotometer (Bio-Tec instruments, Inc., Winooski, VT, USA) and the results expressed as the mean percentages of the total cellular enzyme content released during activation by the corresponding fMLP/CB-activated, drug-free control systems.

### 2.5. Radiometric Assessment of Transmembrane Ca^2+^ Fluxes

Calcium-45 chloride (^45^Ca^2+^, specific activity 18.53 mCi/mg, PerkinElmer-NEN Research Products, Boston, MA, USA) was used as tracer to label the intracellular Ca^2+^ pool and to monitor Ca^2+^ influx in moxifloxacin-treated neutrophils. In the assay of Ca^2+^ influx described below, the radiolabelled cation was used at a fixed, final concentration of 2 µCi/mL, containing 50 µM cold carrier Ca^2+^ (as CaCl_2_) and the final assay volumes were 5 mL containing a total of 1 × 10^7^ neutrophils. The standardization of the procedure used to load the cells with ^45^Ca^2+^ has been described previously [12].

To measure the net influx of ^45^Ca^2+^ into fMLP-activated neutrophils, uncomplicated by concomitant efflux of the radiolabelled cation, the cells were preincubated for 15 min at 37 °C in Ca^2+^-replete HBSS, then pelleted by centrifugation and resuspended to 1 × 10^7^ cells/mL in HBSS containing 250 µM cold Ca^2+^. Pre-loading of neutrophils with cold Ca^2+^ was undertaken to ensure that intracellular Ca^2+^ stores were replete, thereby minimizing spontaneous uptake of ^45^Ca^2+^ (unrelated to fMLP activation) in the influx assay. The Ca^2+^-loaded neutrophils (2 × 10^6^ cells/mL) were then preincubated for 10 min at 37 °C in HBSS containing a final concentration of 50 µM cold, carrier Ca^2+^ in the presence and absence of moxifloxacin (10 µg/mL final). This was followed by the simultaneous addition of fMLP(1 µM) and ^45^Ca^2+^ (2 µCi/mL), or ^45^Ca^2+^ only to control, unstimulated systems. The influx of ^45^Ca^2+^ into fMLP-activated neutrophils was determined 5 min later when influx is complete [9], and the values compared with the uptake of the radiolabelled cation by identically processed unstimulated cells using liquid scintillation spectrometry. Briefly, the cells were washed twice in ice-cold HBSS, followed by lysis of the cell pellets with 0.5 mL of Triton X-100/NaOH (0.1%:0.05 M), addition of scintillation cocktail and detection of the amount of cell-associated radioactivity (counts per minute) using a Tri-Carb-2100TR (Packard, Meriden, CT, USA) liquid scintillation spectrometer. 

### 2.6. Cellular ATP Levels

Measurement of cellular ATP levels was performed to investigate the cytotoxic potential of moxifloxacin for neutrophils. Neutrophils (1 × 10^6^ cells/mL) were incubated in the presence and absence of moxifloxacin (2.5, 5, 10 and 20 µg/mL) for 10 min in a 37 °C waterbath. Following incubation, 20 µL of cell suspension were added into pre-prepared chemiluminometer cuvettes containing 100 µL of nucleotide releasing agent (NRS), which causes release of ATP from the cells, and 30 µL of ATP assay mix dilution buffer (FL-AAM). After vortexing, 20 µL of ATP assay mix was added to the mixture, and chemiluminescence measured using the Lumac Biocounter^®^ 2010M and the results (r.l.u.) converted to nmoles/10^6^ cells using a standard curve.

### 2.7. Mononuclear Leukocytes

Purified human mononuclear leukocytes (MNL) were separated from granulocytes by centrifugation on Histopaque^®^-1077 cushions at 400 g for 25 min at room temperature. The mononuclear leukocyte (MNL) layer was removed and cells were washed with PBS containing ethylene glycol-bis(β-aminoethylether)-*N,N,N’,N’*-tetraacetic acid (EGTA, 1 mM) to prevent aggregation of the cells. After centrifugation at 250 g for 10 min, residual erythrocytes were removed by selective lysis with 0.84% NH_4_Cl for 10 min at 4 °C. The resultant pellet was then washed with PBS/EGTA. The MNLs, which were routinely of high purity and viability (>90%), were then resuspended to 1 × 10^7^ cells/mL in RPMI 1640 tissue culture medium and held on ice until use. Purity of isolated lymphocytes was assessed microscopically and assessment of viability was done bydye-exclusion using 0.1% methylene blue.

### 2.8. Mitogen-Activated Lymphocyte Proliferation Assay

MNL were resuspended to 1 × 10^6^ cells/mL in RPMI 1640 and added to the wells (50 µL of cell suspension *i.e.* 5 × 10^4^ MNL/well) of micro-tissue culture plates together with foetal calf serum (FCS, 10% final) with or without moxifloxacin (2.5–10 µg/well, final) in a final volume of 200 µL/well. The plates were then incubated for 24 h at 37 °C in a humidified CO_2_ incubator (5% CO_2_) before the addition of the T-cell mitogen phytohemagglutinin (PHA 2.5 and 5 µg/mL, final) and incubated for a further 48 h. Proliferation was assessed radiometrically according to the magnitude of uptake of tritiated thymidine (^3^H, specific activity 0.2 µCi/well, PerkinElmer-NEN, Research Products, Boston, MA, USA) for 18 h, into the newly synthesized DNA of the dividing cells. Cells were then harvested on glass fiber filters using the PHD multi-well cell harvester (Cambridge Technology, MA, USA). The disks were dried using methanol, placed in glass vials, followed by the addition of 4 mL scintillation fluid and the amount of radioactivity incorporated into DNA of cells in each well was measured using the liquid scintillation spectrometer.

### 2.9. Analysis of CD25 Expression

The effects of moxifloxacin on the functional responses of lymphocytes were also assessed according to the expression of the surface activation marker CD25 (IL-2αR), which is an alternative method to evaluate T-cell proliferation. Lymphocytes (1 × 10^6^ cells/mL) were resuspended in FCS-supplemented RPMI 1640 in the presence and absence of moxifloxacin (2.5–10 µg/mL) and incubated for 24 h at 37 °C in a CO_2_ incubator (5% CO_2_) before the addition of the mitogen, PHA (2.5 and 5 µg/mL, final). The tubes, which contained a final volume of 2 mL were incubated for a further 24 h, and CD25 was detected flow cytometrically using an anti-CD25 FITC-conjugate. Briefly, 500 µL of cultured lymphocyte suspension were diluted with 500 µL HBSS. The cells were then incubated for 15 min at room temperature in the dark with anti-CD25 FITC monoclonal antibodies (mAb), or an anti-IgG FITC conjugate for detection of nonspecific background staining. The Epics Altra Flow Cytometer (Beckman Coulter, Miami, FL, USA) equipped with a water-cooled coherent Enterprise laser, was used to detect the CD25 positive cells. Expo 32 software (Beckman Coulter) was used to analyze the data. Both the percentage CD25 positive cells and the density of the activation marker expression (mean fluorescence intensity, MFI) were calculated and represented as the normalized mean fluorescence intensity (NMFI) value, which is the percentage of positive lymphocytes multiplied by the corresponding MFI values.

### 2.10. Statistical Analysis

The results of each series of experiments are expressed as the mean values ± the standard error of the mean (SEM). Levels of statistical significance were calculated by paired Student’s *t*-test. P values of ≤0.05 were considered significant. The *n* value indicated in Tables and Figures represent the number of different donors used in each series of experiments.

## 3. Results

### 3.1. Effects of Moxifloxacin on Lucigenin- and Luminol-enhanced Chemiluminescence Responses of Neutrophils Activated with fMLP or PMA

These results for fMLP- and PMA-activated neutrophils are shown in Table 1aand Table 1b for lucigenin- and luminol-enhanced chemiluminescence responses respectively. Moxifloxacin did not affect oxidant (superoxide and oxidants derived from the MPO/H_2_O_2_/halide system) generation by either fMLP- or PMA-stimulated neutrophils. 

**Table 1a:** Effects of moxifloxacin (1–20 µg/mL) on superoxide production by fMLP- or PMA-activated neutrophils.

	Lucigenin-enhanced Chemiluminescence	
System	fMLP-activated	PMA-activated Resting cells
Stimulant only (control)	3891 ± 563	3379 ± 195
Stimulant + moxifloxacin 1 µg/mL	3883 ± 550	3504 ± 188
Stimulant + moxifloxacin 2.5 µg/mL	3967 ± 577	3539 ± 228
Stimulant + moxifloxacin 5 µg/mL	3577 ± 547	3856 ± 340
Stimulant + moxifloxacin 10 µg/mL	3796 ± 472	3456 ± 221
Stimulant + moxifloxacin 20 µg/mL	4211 ± 456	3367 ± 194

The results of 10 different experiments (*n* = 10) with a minimum of two replicates for each system, are expressed as the mean absolute peak values ± SEMs measured 1 and 10 min after the addition of fMLP or PMA respectively (in relative light units and mV/sec). The values for the corresponding unstimulated control systems were 820 ± 58 relative light units and 485 ± 82 mV/sec.

**Table 1b:** Effects of moxifloxacin (1–20 µg/mL) on production of oxidants by the MPO/H_2_O_2_/halide system following activation of neutrophils with fMLP or PMA.

	Luminol-enhanced Chemiluminescence	
System	fMLP-activated	PMA-activated Resting cells
Stimulant only (control)	35898 ± 7256	23938 ± 1946
Stimulant + moxifloxacin 1 µg/mL	33838 ± 6574	22108 ± 1770
Stimulant + moxifloxacin 2.5 µg/mL	33965 ± 6095	21217 ± 1656
Stimulant + moxifloxacin 5 µg/mL	34069 ± 6291	20868 ± 1680
Stimulant + moxifloxacin 10 µg/mL	34691 ± 6325	21040 ± 1816
Stimulant + moxifloxacin 20 µg/mL	34711 ± 4859	19273 ± 1521

The results of three different experiments (*n* = 3) with a minimum of two replicates for each system, are expressed as the mean absolute peak values ± SEMs measured 1 and 10 min after the addition of fMLP or PMA respectively (in relative light units and mV/sec). The values for the corresponding unstimulated control systems were 3142 ± 211 relative light units and 1804 ± 82 mV/sec.

### 3.2. Effects of Moxifloxacin on Elastase Release by fMLP/CB-Activated Neutrophils

**Figure 1:**
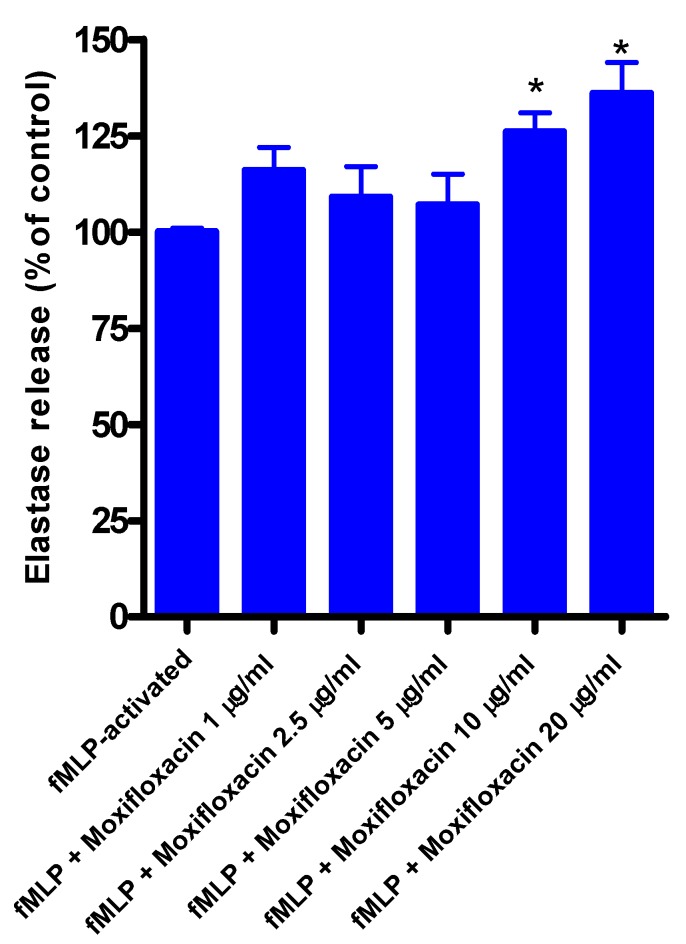
Effects of moxifloxacin on the release of elastase from fMLP/CB-activated neutrophils.

The results are shown in Figure 1. Moxifloxacin at concentrations of 1, 2.5, and 5 µg/mL did not affect the release of elastase by neutrophils measured 30 min after the addition of fMLP/CB, while at concentrations of 10 and 20 µg/mL release of elastase was significantly increased. Moxifloxacin alone did not affect the release of elastase from unstimulated neutrophils. The corresponding values for unstimulated cells (without fMLP/CB) were 112 ± 9, 110 ± 6, 122 ± 3, 117 ± 3 and 110 ± 6 for systems exposed to 1, 2.5, 5, 10 and 20 µg/mL moxifloxacin, respectively (results expressed as the mean percentage of the moxifloxacin-free control system).

The results of four different experiments (*n* = 4) with 5-6 replicates for each system, are expressed as the mean percentages ± SEMs of the corresponding moxifloxacin-free control systems.

### 3.3. Effects of Moxifloxacin on Influx of ^45^Ca^2+^

For these experiments, neutrophils were preloaded with cold Ca^2+^ (to minimize the spontaneous uptake of ^45^Ca^2+^ in the influx assay), transferred to low-(50 µM) Ca^2+^ HBSS, and incubated with moxifloxacin at a fixed, final concentration of 10 µg/mL for 10 min at 37 °C prior to the simultaneous addition of fMLP (1 µM) and ^45^Ca^2+^ (2 µCi/mL). Activation of control, drug-free neutrophils with fMLP resulted in a delayed influx of ^45^Ca^2+^, which occurred after a lag phase of 30-60 seconds. The influx of ^45^Ca^2+^ appeared to be a true consequence of the activation of neutrophils with fMLP, as the unstimulated control did not show a marked increase in intracellular ^45^Ca^2+^ levels. The mean uptakes of ^45^Ca^2+^ by fMLP-activated control and moxifloxacin-treated neutrophils were 526 ± 35 and 570 ± 32 pmol ^45^Ca^2+^/10^7^ cells respectively, measured 5 min after addition of fMLP when influx is complete [12].

### 3.4. Effect of Moxifloxacin on Neutrophil ATP Levels

Neutrophils were exposed to moxifloxacin (2.5–20 µg/mL) for 10 min at 37 °C. The values for untreated cells were 65.2 ± 5.2 nmoles/10^6^ cells while the values for those treated with moxifloxacin (2.5, 5, 10 and 20 µg/mL) were 64.4 ± 5.6, 67.4 ± 7.9, 70.8 ± 5.7, and 64.9 ± 5.7 nmoles/10^6^ cells respectively (*n* = 2 with two replicates for each system). These results indicate that treatment of neutrophils with moxifloxacin at concentrations of up to 20 µg/mL does not affect cell viability.

### 3.5. Effects of Moxifloxacin on Lymphocyte Proliferation and Expression of CD25

**Figure 2:**
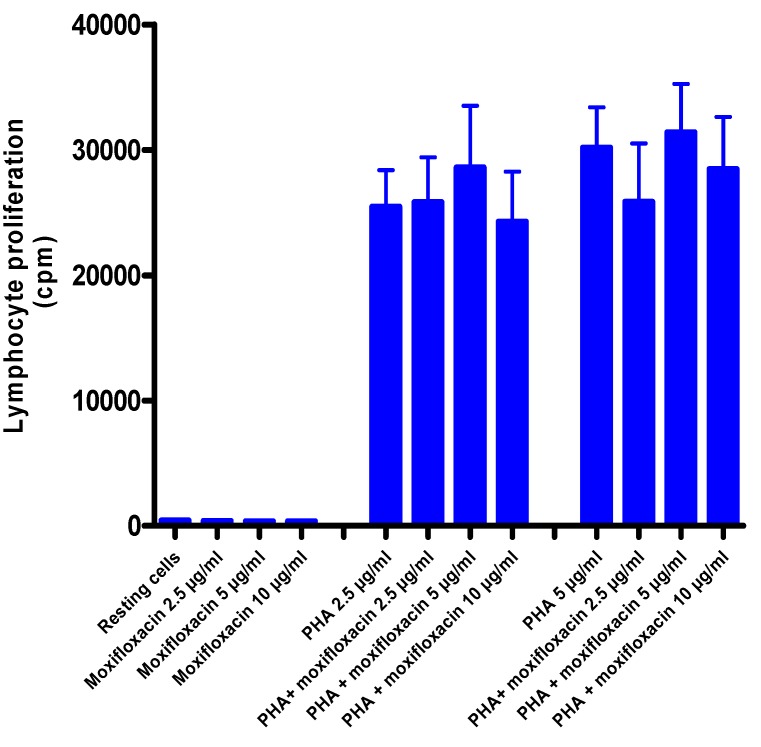
Effects of moxifloxacin on proliferation of PHA-activated MNL.

**Figure 3:**
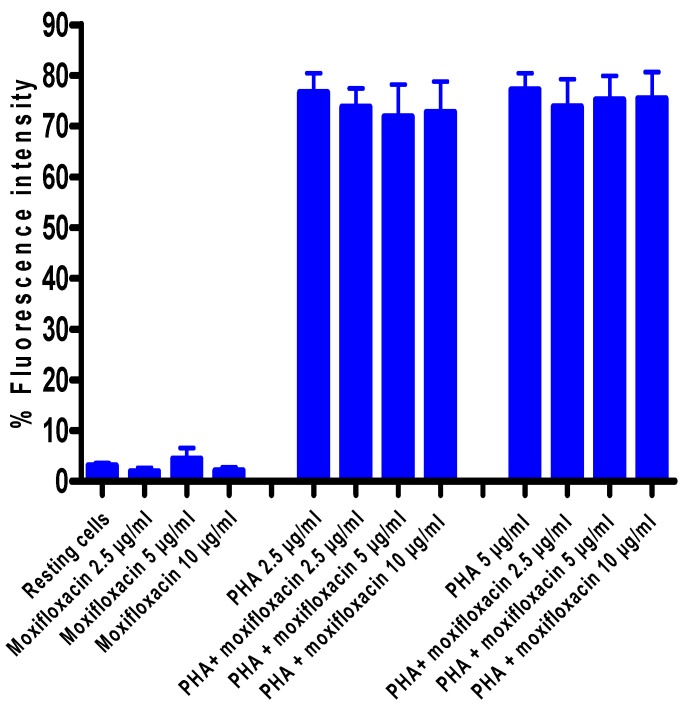
Effect of moxifloxacin on CD25 expression by PHA-activated MNL.

To investigate the effect of moxifloxacin on lymphocyte proliferation, ^3^H-thymidine incorporation into newly synthesized DNA of T-cells was measured after a 24 h treatment of cells with moxifloxacin followed by activation with PHA, while expression of CD25 was measured by flow cytometry. As shown in Figure 2 and Figure 3, moxifloxacin at concentrations of 2.5, 5 and 10 µg/mL did not affect either lymphocyte proliferation or CD25 expression.

The results of five different experiments (*n* = 5) with three replicates for each system, are presented as the mean values in radioactive cpm ± SEMs for uptake of radiolabelled thymidine by unstimulated and PHA-activated MNL in the absence and presence of moxifloxacin.

The results of seven different experiments (*n* = 7) are presented as the mean values ± SEMs for CD25 expression of unstimulated and PHA-activated MNL in the absence and presence of moxifloxacin.

## 4. Discussion

The results of the current study demonstrate that moxifloxacin, at therapeutically-relevant concentrations and above, does not affect the generation of antimicrobial reactive oxygen species by neutrophils exposed to either receptor-dependent or -independent activators of NADPH oxidase. This observation is in agreement with an earlier, unconfirmed study by Fischer and Adam, who observed that treatment of isolated, human blood neutrophils with moxifloxacin at concentrations of up to 100 µg/mL *in vitro* did not affect the phagocytic, respiratory burst, or antimicrobial activities of these cells [7]. Following ingestion of a single 400 mg tablet of moxifloxacin by adult humans, peak serum concentrations of this agent, attained at 2–6 h, are 6.6–10 µg/mL [7,8,9]. Important differences exist between the study reported by Fischer and Adam [7] and the current study. In their study, Fischer and Adam focused on the effects of moxifloxacin, at concentrations of up to 100 µg/mL, on the phagocytic and respiratory burst activities of neutrophils activated with either *Candida albicans* or *Staphylococcus aureus* [7]. In the current study, we have used a chemoattractant (fMLP) and a phorbol ester (PMA) which both utilize different transductional mechanisms from one another, as well as from phagocytic stimuli, to activate NADPH oxidase. We also used procedures which measure a range of reactive oxygen species. In addition, we have investigated the effects of moxifloxacin on fMLP-activated cytosolic Ca^2+^ fluxes, which precede and are a prerequisite for receptor-mediated activation of neutrophils, as well as the effects of the antibiotic on release of elastase.

Treatment of neutrophils with moxifloxacin at concentrations of up to 5 µg/mL did not affect the release of elastase from activated neutrophils. At higher concentrations, however, release of this primary granule protease from chemoattractant-activated neutrophils was modestly, but significantly, increased. Elastase release from activated neutrophils is a Ca^2+^-dependent event [13]. However, no effects of the antimicrobial agent on Ca^2+^ influx following activation of the cells with fMLP were observed, suggesting that moxifloxacin-mediated augmentation of elastase release occurs by a mechanism downstream of Ca^2+^ mobilization. Elastase possesses antimicrobial properties [14,15], and also facilitates transendothelial migration of these cells [16]. If operative *in vitro*, this activity of moxifloxacin may potentiate the protective migratory and antimicrobial functions of neutrophils. On the other hand, however, exaggerated release of elastase poses the potential hazard of inflammation-mediated tissue damage [17]. Importantly, exposure of neutrophils to moxifloxacin at concentrations of up to 20 µg/mL for 10 min (the time course of most experiments) did not affect cell viability as measured by cellular ATP levels, which is also in agreement with the lack of inhibitory effects of this agent on neutrophil functions.

Treatment of isolated, human blood MNL with moxifloxacin at concentrations of up to 10 µg/mL did not affect the proliferative responses of lymphocytes activated by the T-cell mitogen, PHA. Likewise, expression of the IL-2 receptor, CD25, a key event in lymphocyte proliferation, was unaffected by exposure of the cells to moxifloxacin.

In conclusion, exposure of neutrophils and T-lymphocytes, key players in innate and adaptive immune responses respectively, to moxifloxacin at concentrations in the therapeutic range and above, did not impair the protective functions of these cells in our experimental setting. Nevertheless, we do concede the limitations of our study which include possible effects of moxifloxacin, either harmful or beneficial, on other arms of the innate or adaptive immune systems, as well as possible *in vivo* effects, associated with varying pharmacokinetic profiles between individuals and possible immunomodulatory effects of drug metabolites.
